# Study of the Activity of 3-benzyl-5-(4-chloro-arylazo)-4-thioxo-imidazolidin-2-one against Schistosomiasis Mansoni in Mice

**DOI:** 10.1100/2012/520524

**Published:** 2012-05-01

**Authors:** Andréa Cristina Apolinário da Silva, Juliana Kelle de Andrade Lemoine Neves, João Inácio Irmão, Vláudia Maria Assis Costa, Valdênia Maria Oliveira Souza, Paloma Lys de Medeiros, Eliete Cavalcanti da Silva, Maria do Carmo Alves de Lima, Ivan da Rocha Pitta, Mônica Camelo Pessoa de Azevedo Albuquerque, Suely Lins Galdino

**Affiliations:** ^1^Laboratório de Planejamento e Síntese de Fármacos (LPSF), Universidade Federal de Pernambuco, Avenida Profssors Moraes Rego 1265, Cidade Universitária, 50670-901 Recife, PE, Brazil; ^2^Departamento de Medicina Tropical, Universidade Federal de Pernambuco, Avenida Profssors Moraes Rego 1265, Cidade Universitária, 50670-901 Recife, PE, Brazil; ^3^Laboratório de Imunopatologia Keizo Asami (LIKA), Universidade Federal de Pernambuco, Avenida Profssors Moraes Rego 1265, Cidade Universitária, 50670-901 Recife, PE, Brazil; ^4^Departamento de Histologia e Embriologia, Universidade Federal de Pernambuco, Avenida Profssors Moraes Rego 1265, Cidade Universitária, 50670-901 Recife, PE, Brazil

## Abstract

Previous studies conducted with the imidazolidinic derivative 3-benzyl-5-(4-chloro-arylazo)-4-thioxo-imidazolidin-2-one (LPSF-PT05) show outstanding activity against adult *Schistosoma mansoni* worms *in vitro*. In the first phase of this study, *S. mansoni*-infected mice were treated, orally, with 100 mg/Kg of the LPSF-PT05 in three formulations: Tween 80 and saline solution, oil/water (70 : 30) emulsion, and solid dispersion with polyethylene glycol (PEG). In the second phase, three other doses of the LPSF-PT05 in PEG were tested: 3, 10, 30 mg/kg. These treatment regimens significantly reduced the number of recovered worms due to increases in the solubility of the compound in this formulation; the greatest reduction (70.5%) was observed at the dose of 100 mg/kg. There was no changes in the pattern of mature egg compared to immature eggs; however there was a significant increase in the number of dead eggs. Histopathological analysis of liver tissue showed changes in morphological aspects of the hepatic parenchyma with decrease exudative-productive hepatic granuloma stages, although we found no significant differences in IFN-**γ**, IL-4, IL-10, or NO production in response to the specific antigen SEA. The results show the derivative LPSF-PT05 to be a potential candidate in the etiological treatment of schistosomiasis with a possible dampening effect of the granulomatous process.

## 1. Introduction

Schistosomiasis is the second most significant parasitic disease in the world after malaria in terms of socioeconomic and public health importance. It is estimated that 207 million people are infected in 74 countries throughout Latin America, Africa, and Asia and more than 779 million people are at risk of infection, with mortality estimated at up to 280,000 deaths annually in sub-Saharan Africa alone [1–3]. Estimates of the global burden of schistosomiasis range from 1.7 to 4.5 million disability-adjusted life years (DALYs) lost [4–6] or even higher [7].

Chemotherapy is currently the main strategy in use for schistosomiasis control. Praziquantel (2-cyclohexylcarbonyl-1,2,3,6,7,11b-hexa-hydro-4H-pyrazino{2,1-a}isoquinoline-4-one) is the drug of choice for the treatment of schistosomiasis because of its efficacy against all schistosome species [8, 9]. The control of Asian schistosomiasis has relied on large-scale chemotherapy using the praziquantel [10]. However, mass treatment does not prevent reinfection [11]. Data assembled over the past five years suggest that schistosomiasis is reemerging in parts of China [12]. Furthermore, despite the fact that there is not yet clear-cut evidence for the existence of praziquantel-resistant schistosome strains, decreased susceptibility to the drug has been reported in several countries [11]. The reliance on one single antischistosomal drug is alarming and the scientific community has called for research and development of novel and inexpensive drugs against schistosomiasis [13, 14].

The imidazolidines are a broad class of bioactive pentagonal heterocyclic compounds with diverse biological activity [15]. The imidazolidine system has antifungal and antimicrobial properties [16], hypnotic [17], and hypoglycemic [18, 19] effects.

Niridazole, 1-(5-nitro-thiazol-2-yl)-imidazolidin-2-one, has been used over the past century for its schistosomicidal properties. The drug received considerable attention, probably because it was one of the early treatment options to be administered orally [20].

The schistosomicidal properties of imidazolidine derivatives have been demonstrated by *in vitro* studies with adult *S. mansoni* worms [21–25]. The 3-benzyl-5-(4-chloro-arylazo)-4thioxo-imidazolidin-2-one, also known as LPSF-PT05 (CAS Registry Number 197504-87-3) [26], used in this work was synthesized by the Laboratório de Planejamento e Síntese de Fármacos (LPSF) (UFPE) using diazonium ions formed from a phenylamine that acts as an electrophilic reagent and engages with the active hydrogen in position 5 of 3-benzyl-4thioxo-imidazolidine-2-one, yielding the arylazo imidazolidine [27].

Recently, Neves and collaborators [28] demonstrated the schistosomicidal activity *in vitro* of LPSF-PT05 with significant ultrastructural changes induced in worms and less cytotoxic effect on splenocytes than praziquantel. Based on this, the purpose of this study was to investigate the effects of LPSF-PT05, *in vivo*, against adult worms of *S. mansoni.* and also the immunomodulating and histopathological effects of the granulomatous inflammation.

## 2. Materials and Methods

### 2.1. Parasites and Hosts

The BH (BH—Belo Horizonte, MG, Brazil) strain of *S. mansoni* that has been maintained in the laboratory was used throughout this study. The strain was kept after it had passed through *Biomphalaria glabrata* molluscs provided by the Department of Tropical Medicine (Universidade Federal de Pernambuco) and Swiss mice (Mus musculus).

### 2.2. Animals

Swiss Webster mice females were used, average weight 20 ± 2 g and 5 weeks of age, and were bred and maintained at the Laboratório de Imunopatologia Keizo Asami (LIKA) of the Universidade Federal de Pernambuco, Recife, Brazil. Animals were housed in a controlled temperature and light environment and were given water and standard diet a*d libitum*. The experiments were approved by the Federal University of Pernambuco's Animal Experiments Ethics Committee, Process no. 009645/2006-23, in accordance with Law 9605 Article 32 Decree 3179. Art 17.

Mice were infected by exposure to a cercarian suspension of *S. mansoni* with approximately 100 ± 10 cercariae, using the tail immersion technique [29].

### 2.3. Experimental Treatment

Animals previously selected and properly weighed were submitted to a common diet with free access to water before the administration of formulations containing LPSF-PT05. In the first formulation, 1% Tween 80 was used to solubilize LPSF-PT05 in a saline solution (LPSF-PT05-Tween). The second formulation was prepared in an oil/water (70 : 30) emulsion (LPSF-PT05-Emulsion). The third formulation was a solid dispersion containing 10% LPSF-PT05 in the hydrophilic polymer polyethylene glycol (PEG) solubilized in water (LPSF-PT05-PEG).

The administration of the three formulations was done orally, after 49 days of the infection, at a dose of 100 mg/Kg for 5 consecutive days. The solid dispersion containing 10% LPSF PEG-PT05 in three other doses (3, 10, and 30 mg/kg) was administered. The controls groups, free of LPSF-PT05, were submitted to the same testing conditions. At 15 days posttreatment, the animals were euthanized by cervical displacement.

### 2.4. Assessment of Parasitological Criteria

Worms were recovered from the hepatic portal system and mesenteric vessels using the perfusion technique described by Smithers and Terry [30]. The percent of reduction in worm number after treatment was calculated by the method of Tendler and collaborators [31] as follows: % reduction = *C* − *V*/*C* × 100, where *C* is the mean number of parasites recovered from infected untreated animals and *V* is the mean number of parasites recovered from treated animals.

Percentages at each egg developmental stage (oogram pattern), the proportion of eggs at various stages of maturity for the quantitative oogram test, were estimated following the experimental method described by Pellegrino and collaborators [32]. One hundred eggs per oogram were randomly chosen, evaluated by microscopic examination, and classified as dead, immature, or mature for all infected untreated and treated groups.

### 2.5. Culture of Spleen Cells

Spleen cell suspensions were prepared from albino Swiss mice infected with *S. mansoni* and treated with 3, 10, 30, or 100 mg/kg of LPSF-PT05-PEG. The suspensions were depleted of erythrocytes by hypotonic lysis with distilled water and resuspended in RPMI 1640 complete medium containing 5% FCS, 10 mM L-glutamine, penicillin (100 U/mL), and streptomycin (100 *μ*g/mL) (Sigma Chemical, St. Louis, MO, USA). Spleen cells were cultured in 48-well flat-bottom plates (Corning Costar 3548) at 5 × 10^6^ cells per well and incubated at 37°C and 5% CO_2_ for 24 and 72 hours, under stimulation with 20 *μ*g/mL SEA (*Schistosoma mansoni* soluble egg antigen). Supernatants from the cultures were harvested for assessment of cytokine and NO levels. For each experiment, the spleen cells of five mice were pooled.

### 2.6. Measurement of Nitrite Production and Detection of Cytokines

Nitrite (NO_2_^−^) accumulation in 72 h supernatants of cultured cells was used as an indicator of NO production and was determined by the Griess reaction with sodium nitrite as a standard, as previously described (detection limit: 1.56 *μ*M) [33]. Fifty microliters of supernatant were incubated for 10 min, in the dark, at room temperature, with 50 *μ*L of a freshly mixed solution of N-[1-naphthyl]-ethylenediamine (1 mg/mL), sulfanilamide (10 mg/mL), and 5% phosphoric acid in distilled water. The absorbance was measured at 540 nm. Production of IFN-*γ*, IL-10, and IL-4 was measured in supernatants of spleen cell cultures harvested after 24 or 72 hours, using a two-site sandwich enzyme-linked immunosorbent assay (ELISA). Levels of IL-4, IL-10, and IFN-*γ* in culture supernatants were determined using antibody pairs and recombinant cytokines from PharMingen, following the manufacturer's instructions, followed by treatment with streptavidin-peroxidase (Sigma). The reaction was developed using ABTS [(2,2′-azinobis (3-ethylbenzthiazoline-6-sulfonic acid)] (Sigma Chemical, St. Louis, MO, USA) as a peroxidase substrate and read at 405 nm.

### 2.7. Histopathological Evaluation

Tissue samples of livers were removed, fixed immediately in 10% neutral-buffered formalin, embedded in paraffin, and 5 *μ*m sections were stained with Mayer's hematoxylin and eosin [34]. The analysis was performed using a video microscope system (LEICA DMIL microscope, LEICA DFC 280 video-camera). Only transverse sections of tissue samples showing granulomas with visible eggs in the center were analyzed. The granulomas were classified according to Costa-Silva et al. [35]; they were discriminated in two granulomatous stages: exudative-proliferative and involutional. Liver egg granulomas of five mice were counted in five successive low power fields (10x) per each group according to different treatment. 

### 2.8. Statistical Analysis

Results were expressed as mean ± SEM. Data were statistically analyzed by one-way analysis of variance, followed by the Mann-Whitney test for parasitological and immunological studies and Student's *t*-test for histopathological study. Measurements with *P* values ≤ 0.05 were considered significantly different.

## 3. Results

Initially, oral doses of 100 mg/kg of the three formulations of LPSF-PT05 was used to treat mice infected with *Schistosoma mansoni*. The average worm burden was significantly lower than in the control group (*P* < 0.05) when treatment was done with LPSF-PT05-PEG with a mean reduction of worm burden ranging from 19.8% to 70.5%. In groups treated with LPSF-PT05-Tween and LPSF-PT05-emulsion, a reduction rate was of 21% and 40%, respectively (Table 1).

The egg count test, although the absence of any one stage of immature eggs was not seen by 15 days after the end of treatment, we observed that the number of immature eggs was lower in all formulations, with a significant reduction (at *P* < 0.05 compared to its control) with the formulation LPSF-PT05-PEG at a dose of 100 mg/kg. This was inversely proportional to the number of dead eggs, which increased in all formulations and was also significant (*P* < 0.001) in the treatment with the formulation LPSF-PT05-PEG at doses of 10, 30, and 100 mg/kg (Table 1).

To investigate the immunomodulating effects of LPSF-PT05-PEG, IFN-*γ*, IL-4, and IL-10 were quantified in supernatants of spleen cell cultures using sandwich ELISA. The NO production was determined by the Griess reaction. 

**Figure 1:**
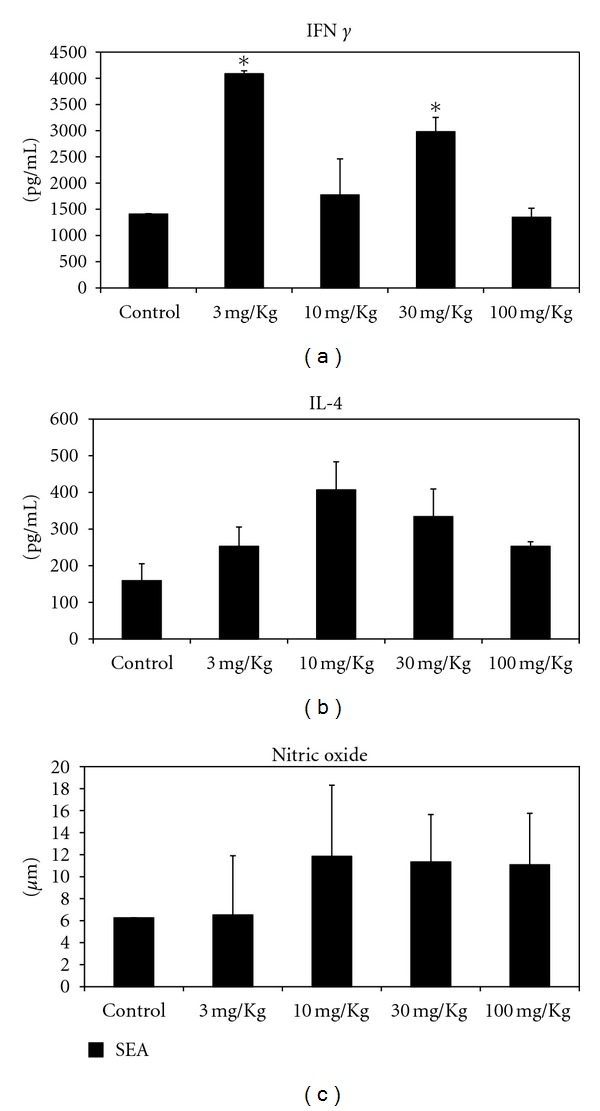
IFN-*γ* (a), IL-4 (b), and nitric oxide (c) production by spleen cells after treatment of *S. mansoni*-infected mice with LPSF-PT05-PEG. *P < 0.001 in comparison with control.

As shown in Figure 1(a), treatment significantly affect IFN-*γ* production in cultures stimulated with the egg antigen in mice treated with 3 mg/Kg and 30 mg/Kg of the drug. IL-4 in SEA-stimulated cultures was higher in cultures from treated animals, but the levels of production of this cytokine were not significant (Figure 1(b)). 

At this stage of infection, nitric oxide production was not affected by the treatment of infected animals. Control and treated mice showed no significant difference in their production of NO. However, the cultures stimulated with SEA showed higher production of nitric oxide for mice treated with doses of 10, 30, and 100 mg/kg, but the difference was not statistically significant (Figure 1(c)). Regarding IL-10 production, this cytokine was below detection level in spleen cell cultures for both control and treated mice. 

Histopathological evaluation of effect of LPSF-PT05-PEG on granulomatous inflammation was measured in H&E stained liver of mice infected by *Schistosoma mansoni*. 

**Figure 2:**
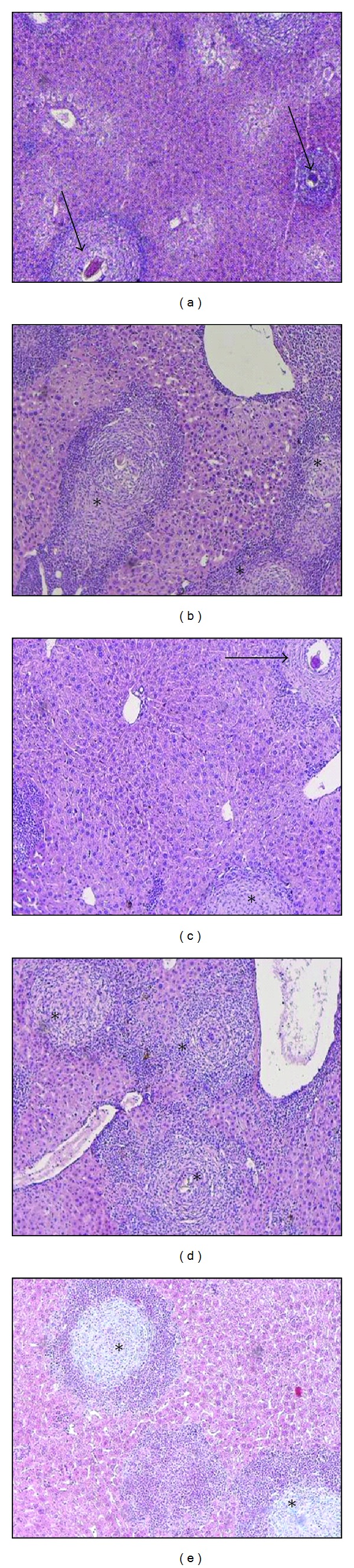
Photomicrographs of granuloma stage in the livers of mice infected by *S. mansoni*. (a) Infected control exhibiting exudative-productive stage granulomas (arrows) with a mature, viable egg in the center of the fibrocellular granulomas. (b) LPSF-PT05-PEG (3 mg/kg per day); micrographs show well-circumscribed small fibrocellular granulomas and marked ovum degeneration (asterisks). (c) LPSF-PT05-PEG (10 mg/kg per day); productive (arrow) and involutional (asterisk) granulomas stage. (d) LPSF-PT05-PEG (30 mg/kg per day). (e) LPSF-PT05-PEG (100 mg/kg per day) involutional stage granulomas (asterisks). H&E, micrographs are at 100x magnification.

The analysis showed that treatment with LPSF-PT05-PEG at doses of 10, 30, or 100 mg/kg per day had a positive effect in reducing the liver damage caused by *S. mansoni* infection, as shown by the reduced number of worms and the downmodulation of granulomatous response (Figure 2), thereby avoiding the development of host pathology. 

Regarding general pathology, the effects of periovular schistosomal granulomas are dynamically similar to those of wound healing, with production of granulation tissue that becomes less vascularized over time, while the fibrous cicatricial tissue becomes more compact and mature. We observed a decrease of the exudative-productive stages in the livers at all concentrations of LPSF-PT05-PEG with a considerably significant decrease at doses of 10 and 100 mg/kg (Figure 2 and Table 2). 

## 4. Discussion

Chemotherapy is the mainstay of schistosomiasis control and is carried out largely through the use of praziquantel. The efficacy of this compound against adult worms of all schistosome species that infect humans has led to its widespread use [8, 9]. The nearly complete reliance on praziquantel for schistosomiasis control may hasten the selection of drug-resistant parasites. The potential for development of resistance to the conventional schistosomicidal drugs has justified the search for new compounds. Several compounds have shown promise for schistosomiasis therapy, for example, artemether, protease inhibitors, 2-(alkylamino)-1-phenyl-1-ethanethiosulfuric acids, and oxadiazoles with emphasis on the 4-phenyl-1,2,5-oxadiazole-3-carbonitrile-2-oxide [36–39]. 

The schistosomicidal effect of imidazolidine derivatives was first reported in 1954 by Luttermoser and Bond [40], who described the activity of 5,5-diphenylhydantoin and 5-(4-chlorophenyl)-5-methylhydantoin against *Schistosoma mansoni* infections in mice. Other studies confirmed modest activity at toxic doses of 5,5-diphenylhydantoin, and 5-(2,4,5-trichlorophenyl) hydantoin showed a potent schistosomicidal effect [41]. 

More recently, evaluation of the schistosomicidal properties of the derivative 3-benzyl-5-(4-chloro-arylazo)-4thioxo-imidazolidin-2-one, or LPSF-PT05, showed higher activity *in vitro* against adult male worms, with 100% mortality after 24 hours of contact at all the concentrations tested. Maximal efficacy against adult female worms was observed after 72 hours. The relationship between the concentration and the effect obtained in a 24-hour period shows a dose-dependent relationship. Electron microscopic observation of the derivative LPSF/PT05 revealed alterations in the integument surface of the worms with the formation of bubbles and peeling, indicating damage to cells; the magnitude of effect was directly related to the duration of exposure [24, 28]. 

This *in vitro* study of LPSF-PT05 confirmed the promising *in vivo* results. However, imidazolidinic compounds present a limitation in their use due to their poor solubility in water. One strategy used to overcome this inconvenience was to create a complex of LPSF-PT05 with the hydrophilic polymer PEG, which was then solubilized in water. This formulation produced a reduction in worm burden of 70.5% compared to a 50% and 30% reduction in worm burden in relation to formulations LPSF-PT05-Tween and LPSF-PT05-emulsion, respectively. The reduced burden of residual worm was also seen in the pattern of egg count, when assessed 15 days after the end of treatment. This showed an increase in the number of dead eggs and decrease in the number of immature eggs which leads us to believe that LPSF-PT05-PEG could have interfered with egg laying by female worms; yet the time of observation adopted in this experiment did not allow us to confirm this suspicion. 

The effect achieved with the formulation of LPSF-PT05-PEG nearly achieved the criterion established by the World Health Organization to identify potential leaders in the development of schistosomicidal compounds, which is defined as a highly active compound that produces a reduction greater than 80% in worm burden after intraperitoneal administration of 100 mg/kg repeated five times in formulations with 10% DMSO [42]. Taking into account the barriers in the process of absorption from oral administration, the LPSF-PT05-PEG, undoubtedly, has potential as a schistosomicide. We believe that adjustments in dosing schedule or even complexion with other adjuvants that promote higher solubility can ensure the schistosomicidal character of this imidazolidic compound. 

Cellular immune response to *S. mansoni* has been intensively studied because of the granulomatous response and fibrosis that occur during pathogenesis. Granulomas play a protective role by sequestering hepatotoxins secreted by eggs [43]; however, they also cause a cell-mediated inflammatory response that results in the pathology of periportal fibrosis [44, 45]. The immune response in *S. mansoni* infection has been shown to be a T-cell-dependent mechanism, where the host initially has a Th1 response against the early stages of the parasite [46]. After deposition of the eggs, the Th2 response increases with IL-4 and IL-5 production [47]. The balance of Th1 and Th2 cytokines is a determining factor in the regulation of pathology and the formation of granulomas and hepatic fibrosis. 

It has been reported that PZQ chemotherapy could modulate humoral and cellular immune responses in individuals infected by *S. mansoni*, probably due to destruction of parasites and releasing of inflammatory stimulating factors such as SEA [48]. In our previous results we observed that praziquantel downregulated the IL-4, modulates IFN-*γ* production, and increased IL-10 production in spleen cells with 120 days of infection (data not shown). 

We expected that LPSF-PT05 could also modulate cytokine production after infect mice treatment. The present study describes the effects of treatment with LPSF-PT05-PEG on the production of cytokines in response to SEA and we found no significant differences in IL-4, IL-10, and nitric oxide production in response to the specific antigen SEA. However, IFN-*γ* production in cultures stimulated with the egg antigen in mice treated with 3 mg/Kg and 30 mg/Kg of the drug was significantly higher in comparison with control group. In spite of these results we did not believe that this IFN-*γ* higher production could affect the evolution of inflammatory response. 

In the histopathological study of *Schistosoma mansoni* infection, the eggs swept into the liver elicit T-cell-dependent responses, which lead to macrophage activation and granuloma formation around the eggs [49]. The severity of the disease is determined by the number of eggs deposited in the tissues and the extent of granuloma formation around them. An important feature of murine schistosomiasis caused by *S. mansoni* is granuloma immunomodulation, that is, the spontaneous downregulation of the response to newly deposited eggs with increased duration of infection [50, 51]. Immunomodulation has been linked to a decrease in T-cell responses to egg antigens and is beneficial to the host in that it limits tissue injury and morbidity [52]. Although we did not detect changes in the profile of IFN-*γ* and IL-4 against the egg antigen, the treated animals sacrificed at 60 days following infection showed changes on morphological aspects of the hepatic parenchyma of mice infected and treated with LPSF-PT05-PEG, at a dose of 100 mg/kg. At this dosage, the involutional granuloma stages limit tissue injury. The decrease in exudative-productive hepatic granuloma stages was observed at all doses, but more so at 100 mg/kg. 

These results together suggest LPSF-PT05-PEG at a dose of 100 mg/Kg as a potential candidate for use in schistosomiasis treatment. At this dose, we found a toxic effect to the worms and an attenuation of granuloma possibly via immunomodulatory properties. Our next concern is to prepare new formulations that can ensure greater solubility of the compound at even lower doses, which would be ideal, since it would minimize the occurrence of side effects and also evaluate the activity of PSF-PEG-PT05 on granulomatous reaction in the chronic phase of schistosomiasis.

## Figures and Tables

**Table 1:** Effect of different formulations of LPSF-PT05 on worm burdens and oogram patterns in experimentally infected mice harbouring adult *S. mansoni* (BH strain).

Group	Dosing protocols (mg/Kg × day)	Worm reductions		% egg developmental stages		
		Total worms	Total worms reduction%	Total Immature	Mature	Dead
Control (7)		24.60 ± 16.29	—	58.88 ± 14.19	27.80 ± 10.18	13.33 ± 4.40
LPSF-PT05-Tween (7)	100 × 5	19.43 ± 7.87	21.0	47.50 ± 7.37	27.93 ± 5.49	24.58 ± 7.97

Control (5)		30.43 ± 12.20		50.72 ± 6.14	29.52 ± 4.16	19.71 ± 5.51
LPSF-PT05-Emulsion (5)	100 × 5	18.25 ± 2.87	40.0	36.10 ± 12.68	31.67 ± 4.35	32.23 ± 14.57

Control (5)		26.50 ± 12.34		57.51 ± 1.17	34.16 ± 3.53	8.330 ± 2.36
LPSF-PT05-PEG (5)	100 × 5	7.80 ± 3.27	70.5^#^	35.34 ± 7.05*	41.41 ± 3.48	23.25 ± 5.01^#^
	30 × 5	14.00 ± 2.08	47.1^#^	49.17 ± 6.06	24.38 ± 4.87	26.46 ± 4.66^#^
	10 × 5	13.80 ± 4.43	47.9^#^	40.59 ± 15.46	33.33 ± 12.02	26.10 ± 3.48^#^
	3 × 5	21.25 ± 3.40	19.8	53.76 ± 5.99	28.33 ± 3.60	16.66 ± 2.35

The values are expressed in means ± SD. *P ≤ 0.05, ^#^ *P* ≤ 0.01 in comparison to control.

Numbers in brackets represent the numbers of mice.

**Table 2:** Hepatic granulomas of mice infected by *S.mansoni* and treated with LPSF/PT05-PEG.

Granuloma stages	Control	3 mg/Kg	10 mg/Kg	30 mg/Kg	100 mg/Kg
Exudative-productive	22.33 ± 4.16	15.66 ± 8.50	8.00 ± 1.70*	20.66 ± 5.80	11.00 ± 1.00*
Involutional	32.33 ± 12.50	43.33 ± 16.20	20.66 ± 7.76	44.00 ± 11.35	29.66 ± 9.29

Animals per groups (*n* = 5). The values are expressed in means ± SD. Comparisons were made by Student's *t*-test (*P ≤ 0.05).
